# Supplementary material for: Community structure and niche differentiation of endosphere bacterial microbiome in Camellia oleifera
Source: Microbiol Spectr. 2023 Oct 17;11(6):e01335-23. doi: 10.1128/spectrum.01335-23 (PMC10715075; doi:10.1128/spectrum.01335-23)
Supplement: Figures S1 to S3, Tables S1 and S2 — Sequencing depth, LDA scores, Faprotax function predictions, α-diversity, enrichment effect. [file spectrum.01335-23-s0001.pdf]

Community structure and niche differentiation of endosphere bacterial microbiome in *Camellia oleifera*

Yan Zhang[1,*]， Chuting Ding[2]， Taoya Jiang[2]， Yuhua Liu[2]， Yang Wu[1], Huiwen Zhou[1]， Lisha Zhang[1]

[1] Institute of Jiangxi Oil-tea Camellia, Jiujiang University, Jiujiang 332005, Jiangxi, China

[2] College of Pharmacy and Life Science, Jiujiang University, Jiujiang City, Jiangxi Province, 332005, China

Supplementary Material

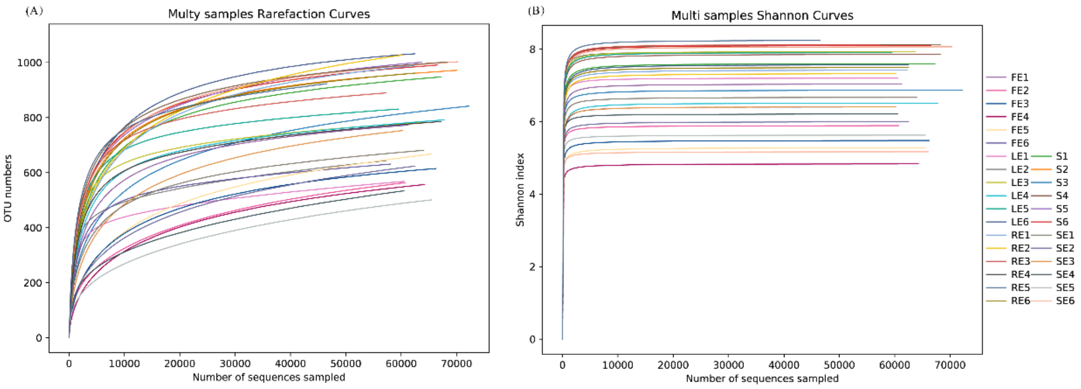

Fig. S1

Normalized rarefaction curves (A) and Shannon-Wienercurves (B) along the number of reads obtained from the different plant compartment of *Camellia oleifera*. The FE, LE, SE, RE and S represent fruit endosphere, leaf endosphere, stem endosphere, root endosphere and bulk soil, respectively.

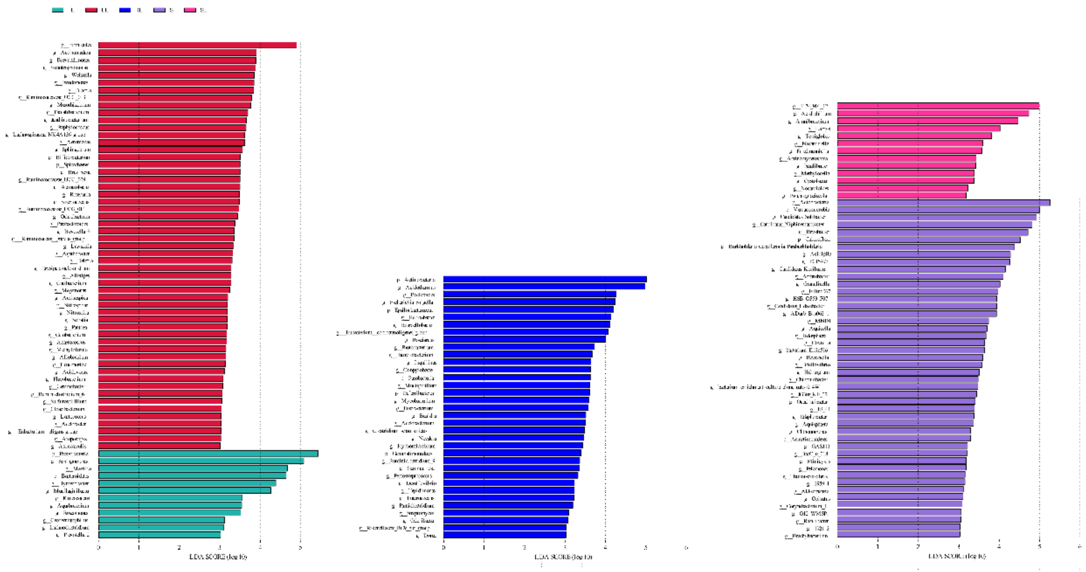

Fig. S2

LDA scores of microbial abundance in different plant compartment of *Camellia oleifera*. The histogram of LDA scores computed for differentially abundant bacterial communities among different *Camellia oleifera* compartments identified with a threshold value of 3.0. The FE, LE, SE, RE and S represent fruit endosphere, leaf endosphere, stem endosphere, root endosphere and bulk soil, respectively.

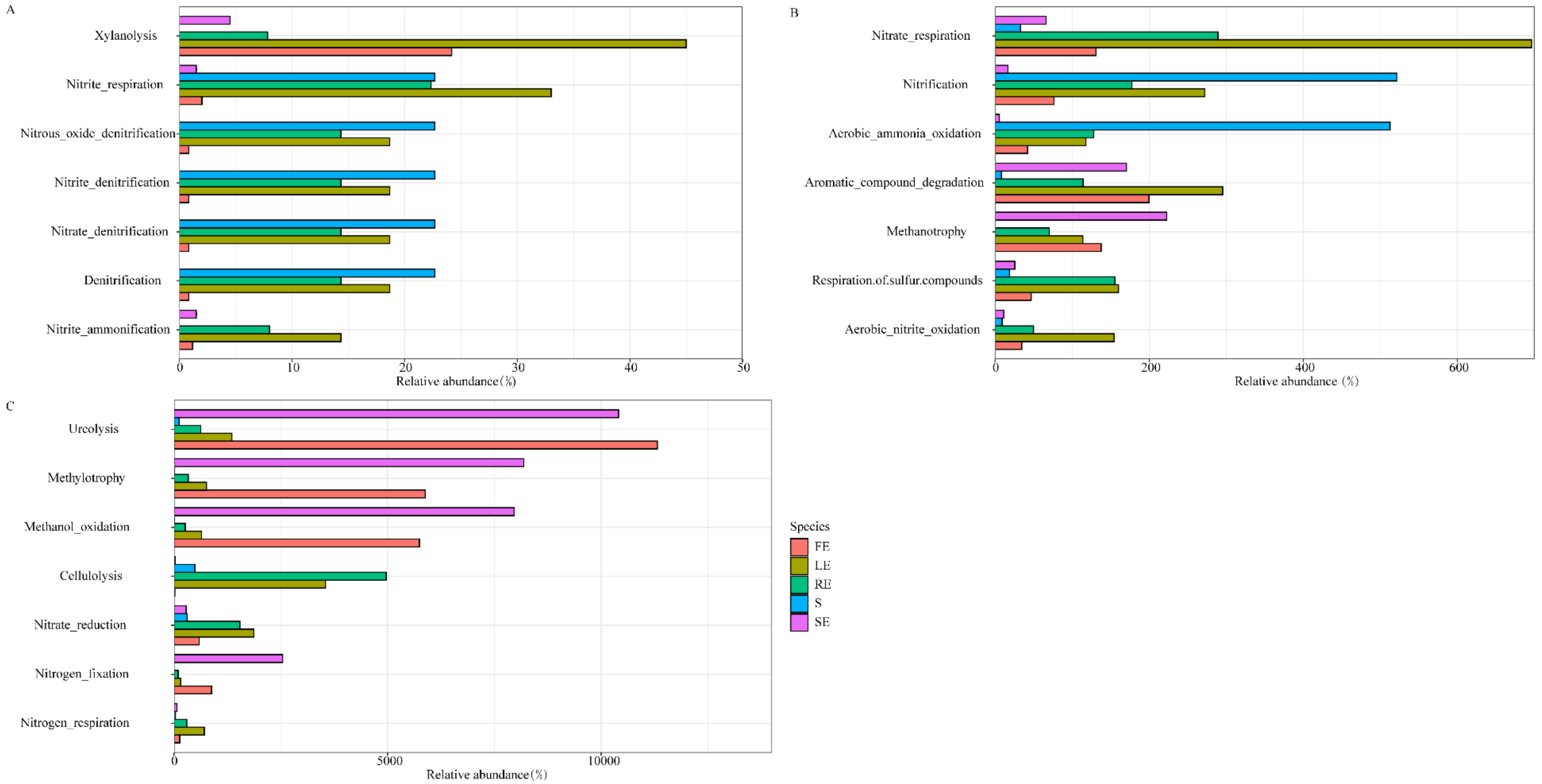

Fig. S3

Faprotax function predictions of the bacterial communities from different plant compartment of *Camellia oleifera*. FE, LE, SE, RE and S represent fruit endosphere, leaf endosphere, stem endosphere, root endosphere and bulk soil, respectively.

Table S1 The α-diversity within each plant compartment. The values represent the mean ± standard error (n = 6). Different letters indicate significant differences ($P < 0.05$) among FE, LE, SE, RE and S. The FE, LE, SE, RE and S represent fruit endosphere, leaf endosphere, stem endosphere, root endosphere and bulk soil, respectively.

	FE	LE	SE	RE	S
ACE	748.8±69.2c	919.9±86.9b	795.4±90.8c	1012.6±93.3ab	1021.8±40.8a
Chao1	782.3±95.3c	914.1±107.9b	763.4±90.6c	1035.4±84.1a	1053.7±30.3a
Simpson	0.947±0.02c	0.979±0.02ab	0.965±0.01b	0.982±0.01a	0.987±0.058a
Shannon	5.82±0.80c	7.51±0.57a	6.01±0.55b	7.60±0.48a	7.70±0.46a

Table S2 Total relative abundances of all phyla and significant effects across plant compartments. Up/down represent the bacterial phyla is significantly enriched/depleted in the latter compartment. FE, LE, SE, RE and S represent fruit endosphere, leaf endosphere, stem endosphere, root endosphere and bulk soil, respectively.

Phylum	S-vs-FE	S-vs-LE	S-vs-SE	S-vs-RE	LE-vs-FE	SE-vs-FE	RE-vs-FE	SE-vs-LE	RE-vs-LE	RE-vs-SE
p_*Acidobacteria*	**DOWN**	**DOWN**	**DOWN**	**DOWN**	**DOWN**	**DOWN**	**DOWN**	NS	NS	**DOWN**
p_*Actinobacteria*	NS	NS	NS	**UP**	**DOWN**	NS	**DOWN**	NS	NS	NS
p_*Armatimonadetes*	**DOWN**	**DOWN**	**DOWN**	**DOWN**	NS	**DOWN**	NS	**DOWN**	NS	**UP**
p_*Bacteroidetes*	**UP**	**UP**	**UP**	**UP**	NS	NS	NS	NS	NS	NS
p_*Deinococcus-Thermus*	**UP**	NS	**UP**	NS	NS	NS	NS	NS	NS	**UP**
p_*Chlamydiae*	**DOWN**	NS	NS	NS	NS	NS	NS	NS	NS	NS
p_*Chloroflexi*	**DOWN**	**DOWN**	**DOWN**	NS	**DOWN**	NS	**DOWN**	**UP**	NS	**DOWN**
p_*Cyanobacteria*	**DOWN**	**DOWN**	**DOWN**	**DOWN**	NS	NS	NS	NS	NS	NS
p_*Deferribacteres*	NS	**DOWN**	**DOWN**	**UP**	NS	NS	**DOWN**	NS	**DOWN**	**DOWN**
p_*Dependentiae*	**DOWN**	NS	**DOWN**	NS	NS	NS	**DOWN**	NS	NS	**DOWN**
p_*Epsilonbacteraeota*	**UP**	**UP**	NS	**UP**	**DOWN**	NS	**DOWN**	**UP**	**DOWN**	**DOWN**
p_*Elusimicrobia*	**DOWN**	**DOWN**	**DOWN**	**DOWN**	**DOWN**	NS	**DOWN**	NS	NS	**DOWN**
p_*Euryarchaeota*	**DOWN**	**DOWN**	**DOWN**	**DOWN**	NS	NS	NS	NS	NS	NS
p_*FCPU426*	**DOWN**	**DOWN**	**DOWN**	**DOWN**	NS	NS	NS	NS	NS	NS
p_*Firmicutes*	**UP**	**UP**	**UP**	**UP**	NS	NS	NS	**UP**	NS	**DOWN**
p_*Fusobacteria*	**UP**	**UP**	**UP**	**UP**	NS	NS	NS	**UP**	NS	**DOWN**
p_*GAL15*	**DOWN**	NS	**DOWN**	NS	**DOWN**	NS	**DOWN**	NS	NS	**DOWN**
p_*Patescibacteria*	**DOWN**	**DOWN**	**DOWN**	**DOWN**	**DOWN**	NS	**DOWN**	NS	NS	NS
p_*Proteobacteria*	**UP**	**UP**	**UP**	**UP**	NS	NS	NS	NS	NS	NS
